# Salvage Surgery for Patients With Local Recurrence or Persistent Disease After Treatment With Chemoradiotherapy for SCLC

**DOI:** 10.1016/j.jtocrr.2021.100172

**Published:** 2021-04-15

**Authors:** Pieter J.M. Joosten, Toon A. Winkelman, David J. Heineman, Sayed M.S. Hashemi, Idris Bahce, Suresh Senan, Marinus A. Paul, Koen J. Hartemink, Max Dahele, Chris Dickhoff

**Affiliations:** a Department of Surgery, Amsterdam University Medical Center, location VUmc Cancer Amsterdam, Amsterdam, The Netherlands; b Department of Surgery, Netherlands Cancer Institute—Antoni van Leeuwenhoek Hospital, Amsterdam, The Netherlands; c Department of Cardiothoracic Surgery, Amsterdam University Medical Center, location VUmc Cancer Amsterdam, Amsterdam, The Netherlands; d Department of Pulmonary Medicine, Amsterdam University Medical Center, location VUmc Cancer Amsterdam, Amsterdam, The Netherlands; e Department of Radiation Oncology, Amsterdam University Medical Center, location VUmc Cancer Amsterdam, Amsterdam, The Netherlands

**Keywords:** Salvage surgery, Small cell lung cancer, Locoregional recurrence, Outcome

## Abstract

**Introduction:**

The role of salvage surgery for patients with locoregional (LR) recurrence or persistent SCLC after radical chemoradiotherapy (CRT) for limited-stage disease is not well established. We evaluated our experience.

**Methods:**

We conducted a retrospective study of consecutive patients who underwent salvage pulmonary resection for LR-recurrent or persistent SCLC between 2008 and 2020 at the Amsterdam University Medical Center.

**Results:**

A total of 10 patients were identified. Median age at initial diagnosis of limited-stage SCLC was 58.5 years (48–71 y). All patients had radical-intent concurrent CRT. Of the 10 patients, 9 were diagnosed with LR-recurrent or persistent disease with a median of 18 months (3–78 y) after CRT. All patients underwent an anatomical radical resection and mediastinal lymph node dissection. No 90-day mortality was recorded. In addition, one patient developed a LR recurrence 7 months after resection. Distant progression was found in three patients at 6, 32, and 61 months after surgery, all of whom subsequently died of progressive SCLC. Median follow-up was 22.5 months (2–86 mos). Disease-free survival was 34 months; overall survival was not reached.

**Conclusions:**

For highly selected patients with LR-recurrent or persistent SCLC after CRT, salvage surgery is feasible and can result in clinically meaningful survival. Such patients should be presented to the multidisciplinary tumor board.

## Introduction

For limited-stage SCLC (LS-SCLC), defined as disease that is limited to the ipsilateral hemithorax and regional lymph nodes, international guidelines recommend combined modality treatment comprising chemotherapy and radiotherapy.^1^ Nonetheless, most surviving patients will develop locoregional (LR) failure.^2^ Second-line treatment generally emphasizes systemic therapy, which in selected patients can deliver meaningful survival gains.^3^^,^^4^ Nonetheless, a subset of patients who progress after radical-intent chemoradiotherapy (CRT) will have limited volume, relatively a localized disease in the thorax. The role of salvage surgery for these patients is not well established.^5^ We therefore evaluated our experience with second-line, salvage surgery for limited-extent LR-recurrent or persistent SCLC after previous CRT.

## Materials and Methods

This retrospective single-center cohort analysis was conducted with the approval of the Amsterdam University Medical Center Institutional Review Board (Research Ethics Committee approval number IRB00002991) and with an appropriate waiver of consent. Patients who underwent pulmonary resection for LR recurrence or persistent disease after CRT for LS-SCLC between January 2008 and December 2020 were identified. We defined salvage surgery as curative (radical)-intent pulmonary resection for LR-recurrent or persistent disease greater than or equal to 12 weeks after the last day of high-dose CRT.^6^ In patients with suspected recurrence on fluorodeoxyglucose positron emission tomography (PET)-computed tomography (CT) scan, pathological confirmation of vital tumor was obtained if possible. Additional endobronchial ultrasound, endoscopic ultrasound, or mediastinoscopy was performed in patients with suspected nodal involvement on PET-CT. Cranial magnetic resonance imaging scan was performed to exclude brain metastases.

Data were extracted from an institutional database, with additional information from other sources (e.g., general practitioner) if necessary. Parameters retrieved included age, sex, comorbidity, physical status, initial clinical TNM staging, pathology, and information on initial diagnosis, staging, and treatment. The eighth edition of the TNM classification for lung cancer was used for staging.^7^ Perioperative data included date of surgery, type of resection, extent of lymph node dissection, inhospital complications, intensive care unit admission, and inhospital length of stay. Additional postoperative information included pathologic TNM staging after salvage surgery, resection margin status, histopathologic diagnosis, site of recurrence after salvage surgery, progression of disease during follow-up, and overall survival (OS).

Statistical analysis was performed using SPSS (version 26, SPSS Inc., Chicago, IL). Median follow-up times were calculated, and disease-free survival (DFS) and OS were analyzed using Kaplan-Meier. DFS was computed from the date of salvage surgery to the date of disease progression (recurrence or metastasis). OS was defined as the time between date of salvage surgery and death from any cause.

## Results

A total of 10 patients who underwent salvage resection for LR-recurrent or persistent disease after radical-intent CRT for LS-SCLC were identified. Median age at time of initial diagnosis of SCLC was 58.5 years (range: 48–71 y), and 80% were of female sex. Detailed patient characteristics are presented in Table 1. All patients had contrast-enhanced thorax CT, PET-CT, and brain magnetic resonance imaging, which were discussed at the multidisciplinary tumor board (MTB) and selected for salvage surgery on the basis of factors that included age, condition and comorbidity, disease-free period after initial CRT, extent of recurrent/persistent disease, and resectability. The MTB included at least two lung cancer surgeons with varying levels of experience.

**Table 1:** Tumor and Treatment Characteristics for Patients Who Underwent Salvage Pulmonary Resection for Recurrent or Persistent SCLC

Pt	Year of Resection	Sex	Age at Initial Diagnosis (y)	cTNM	Histological features	Radiation Dose (Gy)	Second-Line Chemo**t**herapy	Reason for Salvage Surgery	Time Between RT and Surgery (mo)	Mediastinal Evaluation (Presalvage)	r-cTNM	ypTNM
1	2020	F	71	cT4N2	SCLC	66	+	R	23	EBUS	r-cT1aN0	ypT2N1
2	2020	F	58	cT2aN1	SCLC	66	−	R	10	EBUS	r-cT1bN0	ypT1cN0
3	2020	F	59	cT3N1	SCLC	56	−	R	23	EBUS	r-cT2aN0	ypT1cN0
4	2020	F	53	cT4N0	SCLC	N/A	−	R	22	EBUS	r-cT4N0	ypT2N0
5	2018	F	64	cT4N2	SCLC	50	−	R	7	−	r-cT1aN0	ypT1aN0
6	2018	F	66	cT3N2	SCLC/NSCLC	N/A	+	R	80	EBUS + CT biopsy	r-cT3N1	ypT0N0
7	2015	M	58	cT3N0	SCLC	66	−	P	3	CT biopsy	r-cT3N0	ypT0N0
8	2012	F	58	cT2aN1	SCLC	46	+	R	47	EBUS	r-cT1cN0	ypT2aN1
9	2011	F	48	cT4N0	SCLC	45	+	R	22	EUS	r-cT2bN1	ypT2bN0
10	2008	M	64	cT1bN0	SCLC	45	+	R	22	EBUS	r-cT1cN0	ypT2N0

c-TNM, clinical TNM; CT, computed tomography; EBUS, endobronchial ultrasound; EUS, esophageal ultrasound; F, female; M, male; N/A, not available; P, persistent; Pt, patient; R, recurrence; r-cTNM, recurrence-clinical TNM; RT, radiotherapy; ypTNM, pathologic TNM.

At the time of initial diagnosis, 9 of 10 patients had SCLC and one patient had a combination of SCLC and NSCLC. Disease stage at initial diagnosis was clinical stage IA (n = 1), IIB (n = 3), IIIA (n = 3), and IIIB (n = 3). Three patients had pathologically proven N2 disease at the time of initial diagnosis. The median radiotherapy dose was 50 Gray (45–66), concurrent with cisplatin/etoposide (n = 8) or carboplatin/etoposide (n = 2). Prophylactic cranial irradiation was performed in 8 of 10 patients. Median time to diagnosis of LR-recurrent or persistent disease after CRT was 18 months (3–78 mos). Before proceeding to salvage resection, in half of the patients (5 of 10), second-line chemotherapy was initiated after diagnosis of recurrent or persistent disease.

The indication for salvage surgery was LR recurrence in nine patients and persistent disease in one patient. Mediastinal nodal evaluation was performed in 9 of 10 patients (endobronchial ultrasound, n = 7; endoscopic ultrasound, n = 1; and transthoracic biopsy, n = 1). Nodal histopathology was inconclusive in two patients because of fibrosis. The clinical stage of the LR-recurrent/persistent tumor was IA (n = 3), IIA (n = 1), IIB (n = 4), and IIIA (n = 2). Median time between last day of radiotherapy and salvage surgery was 22 months (3–80 mos).

All patients underwent an anatomical pulmonary resection (Table 2). Median duration of surgery was 180 minutes (135–295 min). In five patients, an intrapericardial dissection was performed owing to extensive fibrosis of the hilum or because of centrally located tumor. Bronchial stump buttressing was performed in all patients. Resection was radical in all patients. In two patients who had pathologyical proven recurrence, no vital tumor was found in the resection specimen by the pathologist: one patient had second-line chemotherapy before salvage resection, and one had resection (because of persistent disease) 3 months after the last day of radiotherapy. Median total inhospital stay was 9 days (5–30 d). Inhospital complications occurred in 5 of 10 (50%) patients: three patients developed a postoperative cardiac arrhythmia, of whom one developed a pneumonia, one had prolonged (>5 d) air leakage, and one required rethoracotomy because of respiratory distress shortly after surgery owing to stenosis of the ostium of the right upper lobe. The 30- and 90-day mortality were 0%.

**Table 2:** Surgical Details, Morbidity, Resection Margin, Progression of Disease, and Overall Survival

Pt	Type of Resection	Operating Time (min)	IP Resection	Bronchial Stump Cover	Separate Dissected LN **S**tations	In**h**ospital Complication	Reintervention	Hospital Stay (d)	LP or DP	Time to Progression	Survival (mo)
1	L LUL	180	+	IMF	5/6, 8, 9, 10, 11	AF, pneumonia	−	13	−	−	≥2
2	L LUL	154	−	IMF	5/6, 7, 10	−	−	5	LP	6	≥11
3	L RUL	244	−	IMF	4, 7	AF	−	13	−	−	≥8
4	P L	N/A	+	IMF	4, 10	−	−	7	−	−	≥8
5	L RUL	161	−	IMF	4, 7	−	−	9	−	−	≥34
6	B RUL/RML	220	+	IMF	4, 7	AF	−	9	−	−	≥35
7	L RUL	295	−	ASM	4, 7, 10, 11	Air leakage	−	12	−	−	≥71
8	S (1,2,6)	135	+	Pericardium	4, 7, 8, 11, 10, 12	−	−	6	DP	61	86^a^
9	B RUL/RML	244	+	Omentum	7, 10	Respiratory distress	Rethoracotomy	30	DP	6	12^a^
10	L RUL	144	−	IMF	4, 7, 9, 11	−	−	9	DP	32	33^a^

AF, atrium fibrillation; ARDS, acute respiratory distress syndrome; ASM, anterior serratus muscle; B, bilobectomy; DP, distant progression; EBUS, endobronchial ultrasound; EUS, esophageal ultrasound; IMF, pedicled intercostal muscle flap; IP, intrapericardial; L, lobectomy; LN, lymph node; LP, locoregional progression; LUL, left upper lobe; N/A, not available; P L, pneumonectomy left sided; RML, right middle lobe; RUL, right upper lobe, S, segment resection.

a Died of disease progression.

**Figure 1:**
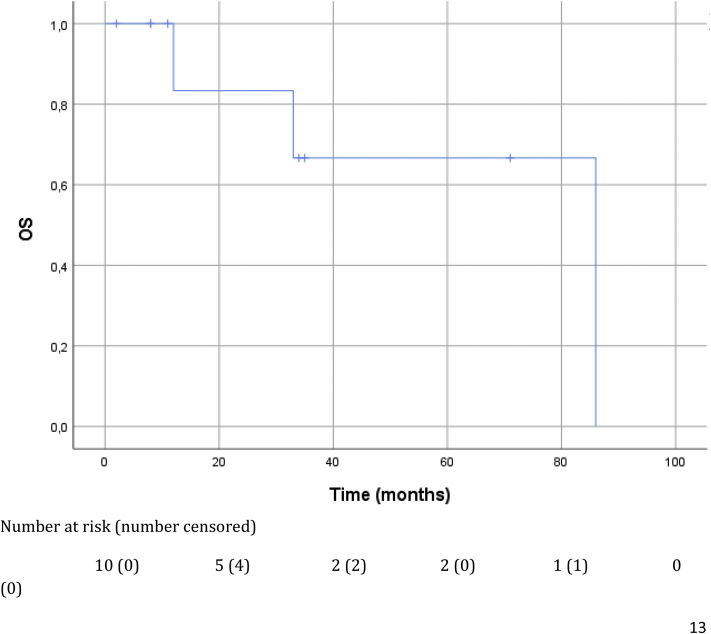
Kaplan-Meier estimates for OS. OS, overall survival.

One patient developed LR recurrence 7 months after surgery. In three patients, distant progression was found at 6, 32, and 61 months postoperatively. During follow-up, three patients, all with distant progression, died. Median follow-up was 22.5 months (2–86 mo) with an estimated median DFS of 34 months, and OS was not reached (Fig. 1).

## Discussion

This is one of the largest single-institution cohorts of salvage surgery for relapsed or persistent SCLC after CRT. In highly selected patients, we report that salvage surgery is feasible, associated with acceptable morbidity and mortality and can result in clinically meaningful survival. These results are in line with those achieved with salvage surgery for patients with recurrent or persistent NSCLC after CRT, who are operated in centers with experience in pulmonary resections after high-dose radiotherapy.^6^^,^^8^, ^9^, ^10^

Approximately one-third of patients with SCLC present with LS-SCLC, and international guideline-recommended therapy for fit patients is combined modality treatment with chemotherapy and concurrent radiotherapy.^3^ Nevertheless, most patients had a relapse (local, distant or both) within 2 years of treatment.^1^^,^^2^ In patients with relapsed SCLC, second-line chemotherapy has been found to increase survival.^3^^,^^11^^,^^12^ In selected patients (fit, with a platinum-sensitive, and therefore delayed, relapse), a Japanese trial has reported that combination second-line chemotherapy may outperform monotherapy; however, considerable hematologic and nonhematologic chemotherapy-related toxicity was reported.^4^ A recent study investigating the role of nivolumab as second-line treatment in relapsed SCLC reported no improvement in survival when compared with second-line chemotherapy.^13^

A subset of relapsed patients will have relatively small-volume, localized thoracic disease. Although the role of salvage surgery in this clinical scenario has not been extensively investigated, the results of our study are congruent with previous reports.^5^^,^^14^ Nakanishi et al.^5^ recently reported favorable survival outcomes in a small carefully selected patient population with ycN0 disease (n = 5). After a median follow-up of 46 months (range: 21–114 mos), four patients had no recurrence of disease and one patient had died owing to disease progression. In our study, in which all patients had pN0 or 1, most of them was disease free at median follow-up of 22.5 months. Although for some patients follow-up is relatively short (≤12 mo in n = 4), DFS is favorable when compared with the results of second-line chemotherapy in unselected patients. Whether the disease is sufficiently localized, technically resectable, and the risks of salvage surgery outweigh the expected outcome with second-line chemotherapy should be considered by the MTB and subsequently discussed with the patient.

In conclusion, surgery has a limited role in SCLC and studies have mainly focused on its role in the initial management of early stage disease. This report suggests that the MTB should consider surgical salvage for highly selected patients with LR-recurrent or persistent SCLC after CRT.
